# Editorial: Targeting the interleukin-1β/interleukin-6/C-reactive protein pathway in clinical medicine - a road map to clinical trial design

**DOI:** 10.3389/fcvm.2025.1644576

**Published:** 2025-06-26

**Authors:** Jan Torzewski, Ahmed Sheriff, Janos G. Filep, Karlheinz Peter, Paul M. Ridker

**Affiliations:** ^1^ Department of Cardiology, Cardiovascular Center Oberallgaeu-Kempten, Clinic Association Allgaeu, Kempten, Germany; ^2^ Medical Department, Charité University Medicine, Berlin, Germany; ^3^ Department of Pathology and Cell Biology, University of Montreal, Montreal, QC, Canada; ^4^ Department of Cardiometabolic Health, Baker Heart and Diabetes Institute, Melbourne, VIC, Australia; ^5^ Heart and Vascular Center, Brigham and Women’s Hospital and Harvard Medical School, Boston, MA, United States

**Keywords:** interleukin-1ß (IL-1ß), interleukin-6 (IL-6), C-reactive protein, cardiovascular disease, clinical trial

**Editorial on the Research Topic**
Targeting the interleukin-1β/interleukin-6/C-reactive protein pathway in clinical medicine - a road map to clinical trial design

The Research Topic “*Targeting the Interleukin-1*β*/Interleukin-6/C-reactive Protein Pathway in Clinical Medicine - A Road Map to Clinical Trial Design*” comprises three original research articles and one review article. Whereas two of the original research articles deal with the idea to complement the predictive value of C-reactive protein (CRP) as a cardiovascular risk marker by adding lymphocyte count to the diagnostic evaluation (Liu et al., Du et al.), the third original research article aims to elucidate the role of Interleukin-1β (IL-1β), Interleukin-6 (IL-6) and CRP in ocular inflammation (Nabaes Jodar et al.). The latter article focuses on the observation that isoforms of pentameric CRP (pCPR), i.e., monomeric CRP (mCRP), may be a better and even more specific marker of intraocular inflammatory conditions in particular and of inflammation in general. In this context, a beautiful review article by Roy et al. on the role of CRP as a potential nexus between inflammation and protein misfolding diseases may well be regarded as the highlight of this Research Topic. The review article further explores the impact of the complex interplay between CRP and its isoforms pCRP, pCRP*, and mCRP in protein misfolding diseases, with a focus on neurodegenerative disease pathogenesis.

Inhibition of IL-1β and IL-6 by specific antibodies has gone far down the line in clinical medicine (1, 2). Concerning chronic application, which is needed for the primary or secondary prevention of cardiovascular disease, costs and infectious complications may present limitations (2). Whereas IL-1β antibodies have been proven to lower cardiovascular events in the absence of any change in cholesterol (3), these agents have been repurposed into oncology given even larger benefits on lung cancer (4). Nonetheless, there is great hope and interest in effects downstream of IL-1β and on IL-6 itself (1, 2, 5). Due to the pivotal role of IL-6 in the human immune system, careful consideration of potential off-target effects caused by chronic IL-6 inhibition is required. The latter issues are currently being investigated in a series of randomized controlled trials (RCTs) which will have a significant impact on our understanding of the role of IL-1β/IL-6/CRP pathway in clinical medicine (1–5). Interestingly, low dose colchicine has recently achieved FDA approval for the prevention of cardiovascular disease and a class IIA recommendation in the American and European Guidelines (6, 7). Colchicine, in addition to tubulin disruption being the primary mechanism of action, may indirectly inhibit NACHT-LRRPYD-containing protein 3 (NALP3) inflammasomes and IL-1β processing and release (6, 7). This may in part explain its effects on cardiovascular disease prevention and CRP levels (7). In contrast to the use of colchicine for chronic stable atherosclerosis, there is controversy related to colchicine in the setting of acute coronary ischemia where trial data to date have been neutral (8) suggesting that “timing the taming of inflammation” may have clinical relevance (9). Gastrointestinal side effects of colchicine and the higher incidence of death from non-cardiovascular causes in the original LoDoCo2 trial (6) remain a matter of concern.

Finally, specific CRP inhibition has become a matter of investigation. Despite huge pharmacological efforts, attempts to specifically inhibit hepatic CRP synthesis have largely failed to reach human application (10). Promising results, mainly in the setting of acute myocardial infarction (AMI), have been achieved with CRP apheresis (11, 12). The scientific community avidly awaits data from the Innsbruck trial re-evaluating the effect of CRP apheresis on the reduction of myocardial infarction size in a randomized controlled setting (https://ichgcp.net/de/clinical-trials-registry; NCT04939805). Depending on the outcome of this trial, a further RCT investigating the effect of CRP apheresis on sound clinical endpoints in AMI is being planned (13).

When additional clinical data become available, a further improvement of the predictive value of CRP by adding additional laboratory parameters (Liu et al., Du et al.) may indeed be needed. Moreover, improvement of CRP inhibiting drugs by interfering with the transformation of pCRP to pCRP* and mCRP (Roy et al.) and thereby increasing their specificity may become a crucial pharmacological path (14, 15).

In summary, this Research Topic may contribute to the understanding of future avenues towards the success of targeting the IL-1β/IL-6/CRP pathway in clinical medicine.
